# The complete mitochondrial genome of the subterranean termite, *Reticulitermes kanmonensis* Takematsu, 1999 (Isoptera: Rhinotermitidae)

**DOI:** 10.1080/23802359.2017.1361363

**Published:** 2017-08-03

**Authors:** Taeman Han, Haechul Park, Jong-Ho Lee, Ki-Jeong Hong, Yongsung Kim, Jongsun Park, Wonhoon Lee

**Affiliations:** a Applied Entomology Division, Department of Agricultural Biology, National Academy of Agricultural Science, Wanju-gun, Jeollabuk-do, Republic of Korea;; b Department of Agricultural Biotechnology, Animal and Plant Quarantine Agency, Gimcheon, Gyeongsangbuk–do, Republic of Korea;; c Department of Plant Medicine, College of Life Science and Natural Resources, Sunchon National University, Suncheon-si, Jeonnam Province, Republic of Korea;; d InfoBoss Co. Ltd, Seoul, Republic of Korea;; e InfoBoss Research Center, Seoul, Republic of Korea;; f Department of Plant Medicine and Institute of Agriculture & Life Science, Gyeongsang National University, Jinju, Republic of Korea

**Keywords:** Isoptera, mitochondrial genome, *Reticulitermes kanmonensis*, rhinotermitidae, Korea

## Abstract

We have determined the mitochondrial genome of *Reticulitermes kanmonensis* Takematsu, 1999. The total length of the *R. kanmonensis* is 16,484 bp with 66.1% A + T content. It consists of 13 PCGs, 22 *tRNA*, 2 *rRNA* genes and an A + T–rich control region. All the protein-coding genes used ATN as start codon. But the stop codons were TAA, TAG, and an incomplete termination codon (T) abutting an adjacent *tRNA* gene. The A + T–rich control region was 1680 bp in length with 70.4% A + T content.

*Reticulitermes kanmonensis* Takematsu (1999) is an economically important termite species in Japan. It has been responsible for damaging traditional wooden houses or cultural properties in Japan (Takematsu 1999). In Korea, the distribution of this species was firstly reported in 2014 (Lee et al. 2015). Although there are some taxonomic studies about this termite (Takematsu 1999; Lee et al. 2015), there is no available information for the mitogenome of this species.

The present study reported the complete mitogenome sequences of *R. kanmonensis*. Specimens were collected from Wanju-gun, Jeollabuk-do, Korea, in April 2014. Voucher specimens (Coll#140428WH08) were deposited at the Insect Collection, Gyeongsang National University, Korea. Total genomic DNA was extracted from the soldier of *R. kanmonensis*. The mitogenome was determined by sequencing with Illumina HiSeq4000, Macrogen, Inc., Korea. Raw sequences were filtered by Trimmomatic and aligned with mitogenome of *R. speratus kyushuensis* (Lee et al. 2017). The mitogenome of *R. kanmonensis* is 16,484 bp in size and its gene content and organization were identical with other *Reticulitermes* species, presenting 13 PCGs, 22 *tRNA* genes, 2 *rRNA* genes, and an A + T–rich control region. The N chain coded 14 gene, including 8 *tRNA* genes (*tRNAGln*, *tRNACys*, *tRNATyr*, *tRNAPhe*, *tRNAHis*, *tRNAPro*, *tRNALeu*(CUN), and *tRNAVal*), four PCGs (*ND5*, *ND4*, *ND4L*, and *ND1*), and two *rRNA* genes (*lrRNA* and *srRNA*). The rest 23 genes were coded by the J chain.

The overall sequences in the mitogenome of *R. kanmonensis* were A + T–biased (the G + C content was 33.9%), as commonly observed in insect mitogenomes. The PCGs region and A + T–rich region accounted for 64.5% AT and 70.4% AT, respectively. *R. kanmonensis* had the typical 22 *tRNA* genes throughout the entire mitogenome. All of them, except that *tRNASer*(AGN) lacked the dihydrouridine (DHU) stem, showed the typical clover-leaf secondary structure. Thirteen open reading frames of *R. kanmonensis* protein-coding sequences had typical ATN initiation codon. Nine genes had a complete TAA termination codon and two genes (*ND1* and *ND2*) had a complete TAG termination codon. While two genes (*COX2* and *ND5*) showed an incomplete termination codon (T) abutting an adjacent *tRNA* gene, which is commonly observed in metazoan animals.

Mitogenome of *R. kanmonensis* also included intergenic spacers and overlapping regions in common with other termite species. The intergenic spacer sequences were spread on 18 regions ranging in size from 1 to 19 bp, and the overlapping sequences varied from 1 to 19 bp in 6 areas. The A + T–rich control region of *R. kanmonensis* located between *srRNA* and *tRNAIle* with 1680 bp long and 70.4% A + T content.

Nucleotide sequences of 13 PCGs from 13 closely related species were analyzed to investigate phylogenetic relationships with *R. kanmonensis*. Phylogenetic analysis was performed using maximum likelihood method with 1000 bootstrap replications with PhyML 3.0 (Guindon et al. 2010). The phylogenetic tree revealed that *Reticulitermes* was a sister group to *Coptotermes*, and *R. kanmonensis* and *Reticulitermes flaviceps* form one clade (Figure 1). Our study of *R. kanmonensis* will be a useful research for understanding the classification and status in Rhinotermitidae.

**Figure 1.**
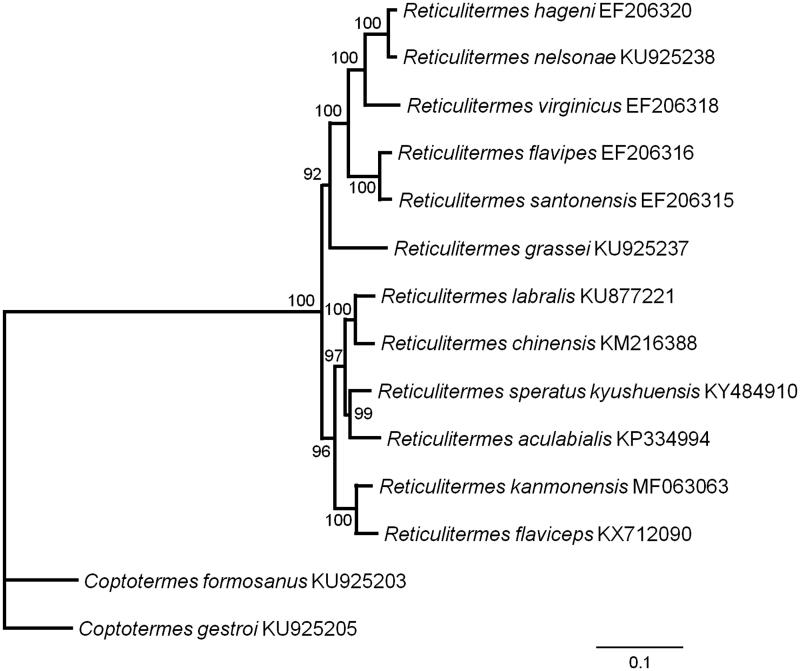
Maximum likelihood estimation of the phylogenetic relationships of two genera *Reticulitermes* and *Coptotermes* based on the nucleotide sequence of 13 PCGs in the mitochondrial genome. The numbers beside the nodes are percentages of 1000 bootstrap values. The *Coptotermes formosanus* and *Coptotermes gestroi* were used as an outgroup. Alphanumeric terms indicate the GenBank accession numbers.

## Nucleotide sequence accession number

The complete mitochondrial genome sequence of *R. kanmonensis* has been assigned GenBank accession number MF063063.
